# Degradation modeling of poly-l-lactide acid (PLLA) bioresorbable vascular scaffold within a coronary artery

**DOI:** 10.1515/ntrev-2020-0093

**Published:** 2020-12-12

**Authors:** Shengmao Lin, Pengfei Dong, Changchun Zhou, Luis Augusto P. Dallan, Vladislav N. Zimin, Gabriel T. R. Pereira, Juhwan Lee, Yazan Gharaibeh, David L. Wilson, Hiram G. Bezerra, Linxia Gu

**Affiliations:** School of Civil Engineering and Architecture, Xiamen University of Technology, Xiamen, Fujian, 361024, China; Department of Biomedical and Chemical Engineering, Florida Institute of Technology, Melbourne, FL 32901, United States of America; National Engineering Research Center for Biomaterials, Sichuan University, Chengdu, 610064, China; Cardiovascular Imaging Core Laboratory, Harrington Heart & Vascular Institute, University Hospitals Cleveland Medical Center, Cleveland, OH 44106, United States of America; Department of Biomedical Engineering, Case Western Reserve University, Cleveland, OH 44106, United States of America; Interventional Cardiology Center, Heart and Vascular Institute, University of South Florida, Tampa, FL 33606, United States of America; Department of Biomedical and Chemical Engineering, Florida Institute of Technology, Melbourne, FL 32901, United States of America

**Keywords:** bioresorbable vascular scaffold, stent, poly-l-lactide acid, degradation, coronary artery, finite element method, percutaneous coronary intervention

## Abstract

In this work, a strain-based degradation model was implemented and validated to better understand the dynamic interactions between the bioresorbable vascular scaffold (BVS) and the artery during the degradation process. Integrating the strain-modulated degradation equation into commercial finite element codes allows a better control and visualization of local mechanical parameters. Both strut thinning and discontinuity of the stent struts within an artery were captured and visualized. The predicted results in terms of mass loss and fracture locations were validated by the documented experimental observations. In addition, results suggested that the heterogeneous degradation of the stent depends on its strain distribution following deployment. Degradation is faster at the locations with higher strains and resulted in the strut thinning and discontinuity, which contributes to the continuous mass loss, and the reduced contact force between the BVS and artery. A nonlinear relationship between the maximum principal strain of the stent and the fracture time was obtained, which could be transformed to predict the degradation process of the BVS in different mechanical environments. The developed computational model provided more insights into the degradation process, which could complement the discrete experimental data for improving the design and clinical management of the BVS.

## 1 Introduction

The permanent implantation of stents, with or without carrying drugs, are permanently caging the vessel and preventing physiologic vasomotor function, which have been associated with undesirable outcomes such as restenosis and thrombosis [1]. Bioresorbable vascular scaffolds (BVS) were developed to overcome the aforementioned limitations [2]. The BVS, also referred to as bioresorbable stent or biodegradable stent, is expected to serve as a temporary scaffold for a period of 6–12 months to allow the artery remodeling and completely reabsorbed within 36 months [3]. The choices of bioresorbable materials as well as structural designs were acknowledged to impact the degradation behavior of BVS [4,5]. Bioresorbable materials of the choice include magnesium-based alloys, pure iron or iron-based alloys, and polymers [6]. Among the several scaffolds developed, the Absorb BVS (Abbott Vascular, Santa Clara, CA), made of poly-l-lactide acid (PLLA), was the first FDA-approved device. It has showed appropriate radial strength and degradation resistance *in vitro* [5,7]. However, its long-term advantages over metallic drug-eluting stents have not been clearly shown [8]. Although the Absorb BVS device was withdrawn from the market, the next-generation BVS systems are currently under development [9,10]. Detailed quantifications of the degradation mechanics could be essential to regulate its degradation process for better clinical outcomes.

Finite element method (FEM) has been widely used to optimize stent design and delineate the interaction between various stents and vessels [11,12], which has been recommended as one part of stent design as stated in the FDA guideline [13]. There are sparse degradation models of the PLLA with a focus on its mechanical integrity. Continuum damage mechanics approach was adopted to model the degradation behavior of the polymer, which is regulated by a scalar variable as the function of time and strain [14,15]. Soares et al. provided a general degradation model of the PLLA with consideration of its hyperelastic mechanical behavior [14]. The degradation was regulated by adjusting the material coefficients. Wang et al. showed that a linear elastic, perfectly plastic constitutive model of the PLLA stent captured its crimping and expansion behavior [16]. However, no degradation was considered. Further, Qiu et al. reported that the yield strength of the linear elastic, perfectly plastic PLLA stent reduced with time during the degradation process [17]. The crimping behavior measured at different time points was captured using the corresponding simulations with adjusted yield strength only. The material softening approach was also adopted for describing the degradation behavior of magnesium stents, in which both the stiffness and yield strength was reduced during the degradation process [18]. On the contrary, Luo et al. showed in their experiments that elastoplastic behaviors of PLLA materials, such as the stiffness and yield strength, exhibited minimal variations during the degradation process, but the fracture strain reduced with the degradation time [15]. This degradation model was implemented and applied to simulate the stent expansion and fatigue. However, the dynamics interaction between the PLLA stent and artery during the degradation process is lacking.

The goal of this work is to delineate the dynamic interactions between the Absorb GT1 BVS and the artery wall during the degradation process. The Absorb PLLA stent was constructed to be implanted in a stylized artery. A strain-driven degradation model is adopted and recalibrated based on the published experimental data [15]. The degradation model was then implemented in the commercial finite element codes Abaqus (Dassault Systèmes Simulia Corp.). The crimping and expansion of the PLLA stent are captured, followed by the heterogeneous degradation considering the interaction between the stent and the artery. The predicted results in terms of mass loss and fracture locations were compared with the documented experimental data [15]. The maximum principal stress/strain distributions, the lumen diameter, and the structural degradation were quantified for a better understanding of the degradation process of the PLLA stent within an artery.

## 2 Materials and methods

### 2.1 Geometry

**Figure 1:**
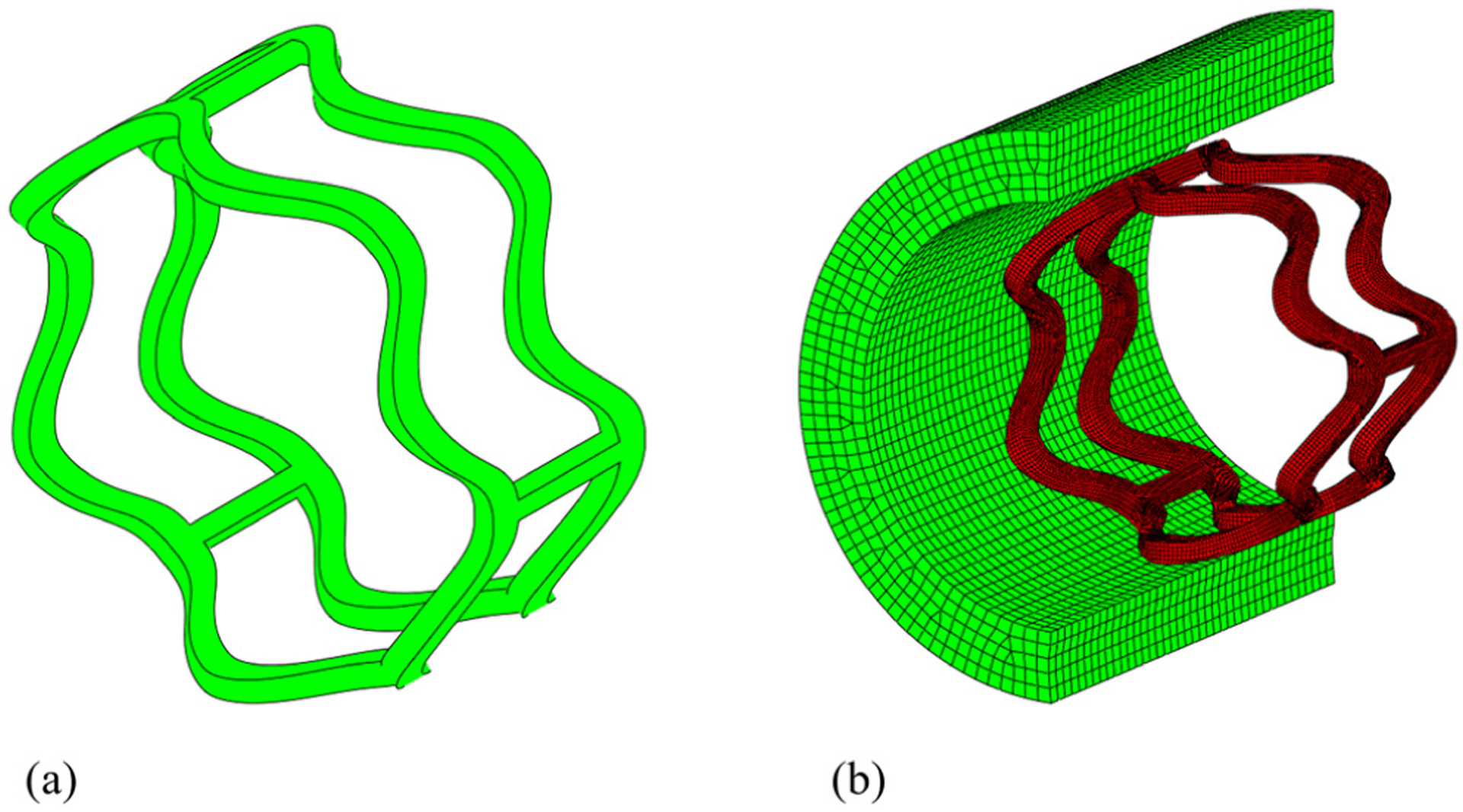
(a) Representative segment of PLLA stent. (b) Finite element model of PLLA stent segment in an artery.

A representative segment of a Absorb GT1 Bioresorbable PLLA stent (Figure 1a) is constructed, with the outer diameter of 3.5 mm and thickness of 0.15 mm [19]. This segment is composed of two curved rings connected by three straight links (Figure 1a). The curved ring has six evenly distributed peaks with the peak-to-valley height as 0.5 mm. The width of the ring strut is 0.2 mm and link strut 0.12 mm. It was used to scaffold a cylindrical artery with an inner diameter of 3mm, thickness of 0.5 mm, and length of 4mm (Figure 1b). The stent segment was discretized into 32,040 brick elements (C3D8R) with an element size of 0.03 mm. The artery was meshed with 23,200 brick elements (C3D8R) with an element size of 0.1 mm [20].

### 2.2 Material properties

**Figure 2:**
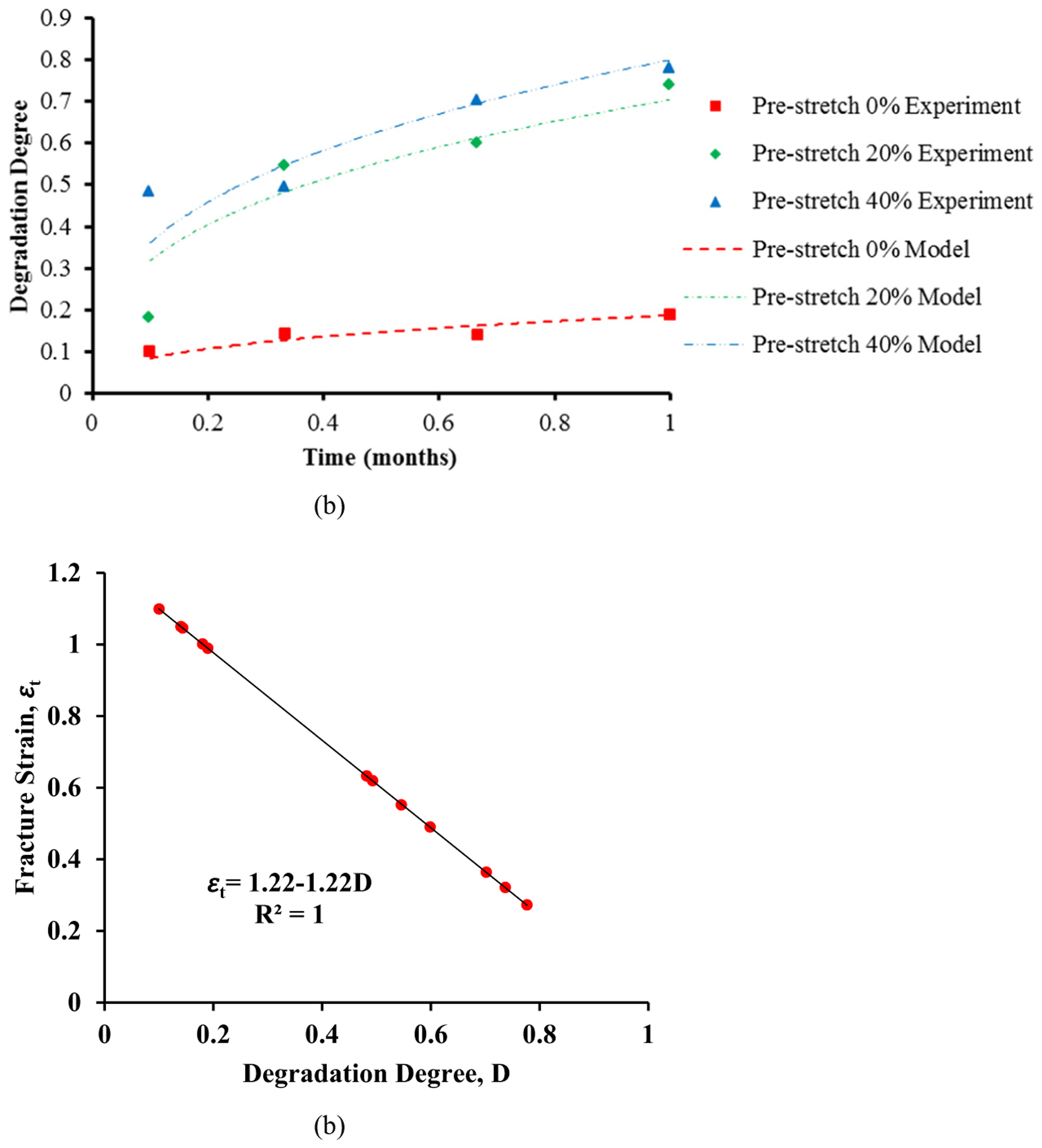
Degradation model of the PLLA material. (a) Degradation degree depends on the maximum principal strain and time. (b) Fracture strain reduced with a larger degradation degree.

The PLLA stent has the Young’s modulus of 1.64 GPa, the Poisson’s ratio of 0.3, and the yield strength of 44 MPa [15,21]. We adopted the degradation constitutive model fitted to the published experimental data [15]. Briefly, the PLLA strips with different tensile strains of 0, 0.2, and 0.4 were exposed to the degradation environment of phosphate buffered saline. Uniaxial tensile test was then conducted at different time point: 3 days, 10 days, 20 days, and 30 days. The stiffness and fracture strains were obtained. It is clear that the fracture strain of PLLA materials decreased during the degradation process [15]. The level of degradation, i.e., change of the fracture strain, has been represented using a degradation degree *D* as:

(1) $$D=\left(\varepsilon_{0}-\varepsilon_{t}\right)/\varepsilon_{0},$$

where *ε*_0_ is the initial fracture strain of the material before degradation and *ε*_*t*_ is the current fracture strain at various degradation states. The degradation degree *D* is 0 when there is no degradation (*ε*_*t*_ = *ε*_0_) and 1 when the material point was completely degraded (*ε*_*t*_ = 0). The evolution of degradation degree *D* depends on the degradation time and strain state, as described below:

(2) $$D\;(\varepsilon ,t)=a\;\left(b+c\times \varepsilon^{n}\right)\;\times \;t^{m},$$

where *ε* is the pre-stretch strain (also the maximum principal strain applied on the PLLA strips), and *t* is the degradation time in days. We obtained the five material constants (*a*, *b*, *c*, *m*, and *n*) using the least squares fitting, as listed in Table 1. The degradation constitutive model and the published experimental data are shown in Figure 2a. A negative linear relationship exists between the degradation degree and the fracture strain (Figure 2b):

(3) $$\varepsilon_{t}=1.22-1.22D.$$

It was then estimated that the initial fracture strain is 1.22 at the degradation degree *D* of 0. The fracture strain decreased to 0 when the degradation degree *D* reached to 1.

A subroutine VUSDFLD was developed in Abaqus/Explicit to capture the degradation behavior of the PLLA stent. The degradation degree is prescribed as a state variable, which is a function of time and the maximum principal strain at each material point (equation (2)). The degradation degree will be updated based on the strain level at each time step. The fracture strain of each element will be updated based on the degradation degree of the material (equation (3)). Once the maximum principal strain decreased to lower than the fracture strain, the fracture will be initiated in the PLLA stent using the element death technique.

The hyperelastic isotropic constitutive model was adopted to describe the nonlinear mechanical behavior of the artery, with a reduced polynomial strain energy density function *U* as:

(4) $$U=C_{10}\left({\bar{I}}_{1}-3\right)+C_{20}{\left({\bar{I}}_{1}-3\right)}^{2}+C_{30}{\left({\bar{I}}_{1}-3\right)}^{3}\;+C_{40}{\left({\bar{I}}_{1}-3\right)}^{4}+C_{50}{\left({\bar{I}}_{1}-3\right)}^{5}+C_{60}{\left({\bar{I}}_{1}-3\right)}^{6},$$

where *Ī*_1_ is the first invariant of the Cauchy–Green tensor: the material constants were adopted from the study by Wu et al., as listed in Table 2 [22].

### 2.3 Boundary conditions and loading steps

The crimping, relaxation, expansion, and recoil of the PLLA stent were simulated before the degradation. Specifically, a radial displacement on the outer surface of the stent was used for stent crimping to an outer diameter of 2.5 mm. Next, the displacement load was removed for the stent relaxation at its crimped state. Following this, the stent expansion in the artery was implemented to reach a diameter of 3.5 mm. The stent recoiled after the unloading, i.e., the withdrawal of the expansion balloon.

After the aforementioned stenting procedure, the degradation process started. It will stop till strut fracture was observed. The Rayleigh damping coefficient of 8,000 was used for the whole model to minimize the dynamic effects, avoid ambiguous displacement fluctuations by energy dissipation [23], and maintain an average ratio of kinetic to internal energy below 5%.

## 3 Results

**Figure 3:**
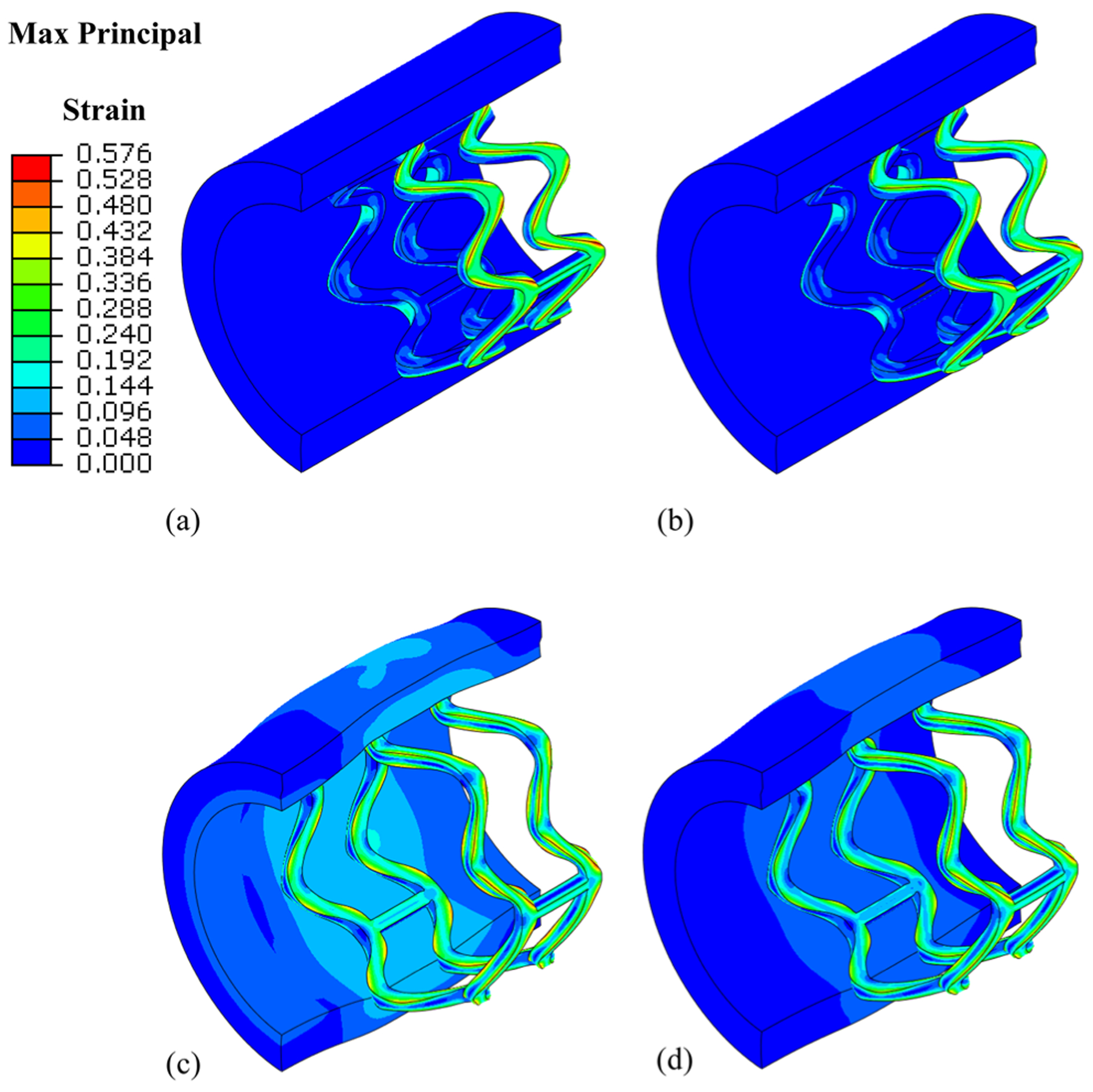
Stenting procedure before degradation. (a) The stent was crimped to an outer diameter of 2.5 mm. (b) The stent relaxed to an outer diameter of 2.65 mm at its crimped state. (c) The stent was fully expanded to an outer diameter of 3.5 mm. (d) The stent recoiled back with an outer diameter of 3.28 mm (only half of the vessel was shown for clear illustration).

The stenting procedure of the PLLA stent is shown in Figure 3. It is clear that the crimping and relaxation of the PLLA stent induced residue stress/strain in the stent. The stent was expanded to 3.5 mm and recoiled back to 3.28 mm, resulting in a recoil rate of 6.3%. Higher strain concentrated at the outer surface of the U-bends, which may indicate a larger degradation rate at these locations.

**Figure 4:**
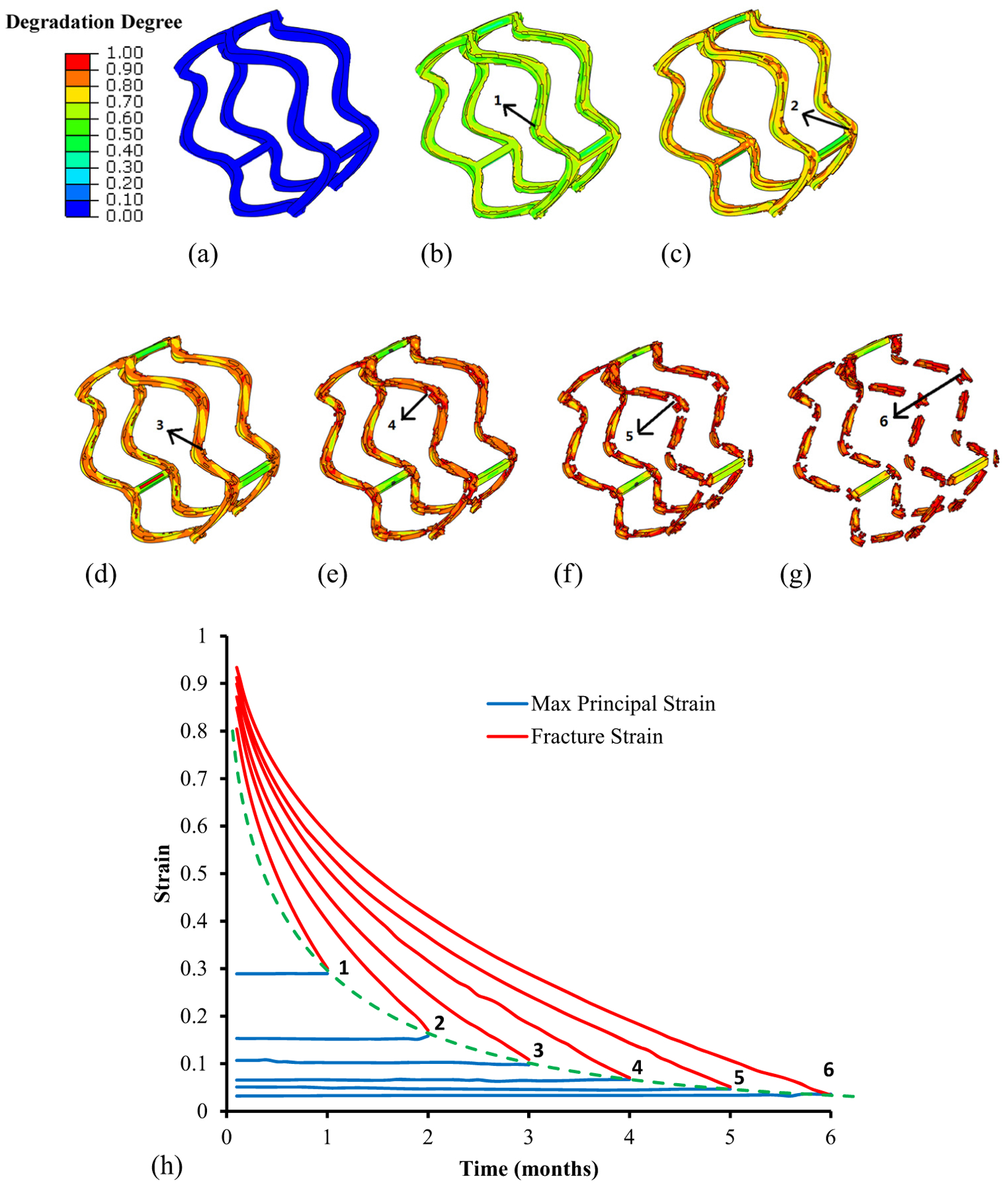
Contour of degradation degree at (a) 0 month, (b) first month, (c) second month, (d) third month, (e) fourth month, (f) fifth month, and (g) sixth month. (h) Maximum principal strain versus fracture strain at six representative elements

**Figure 5:**
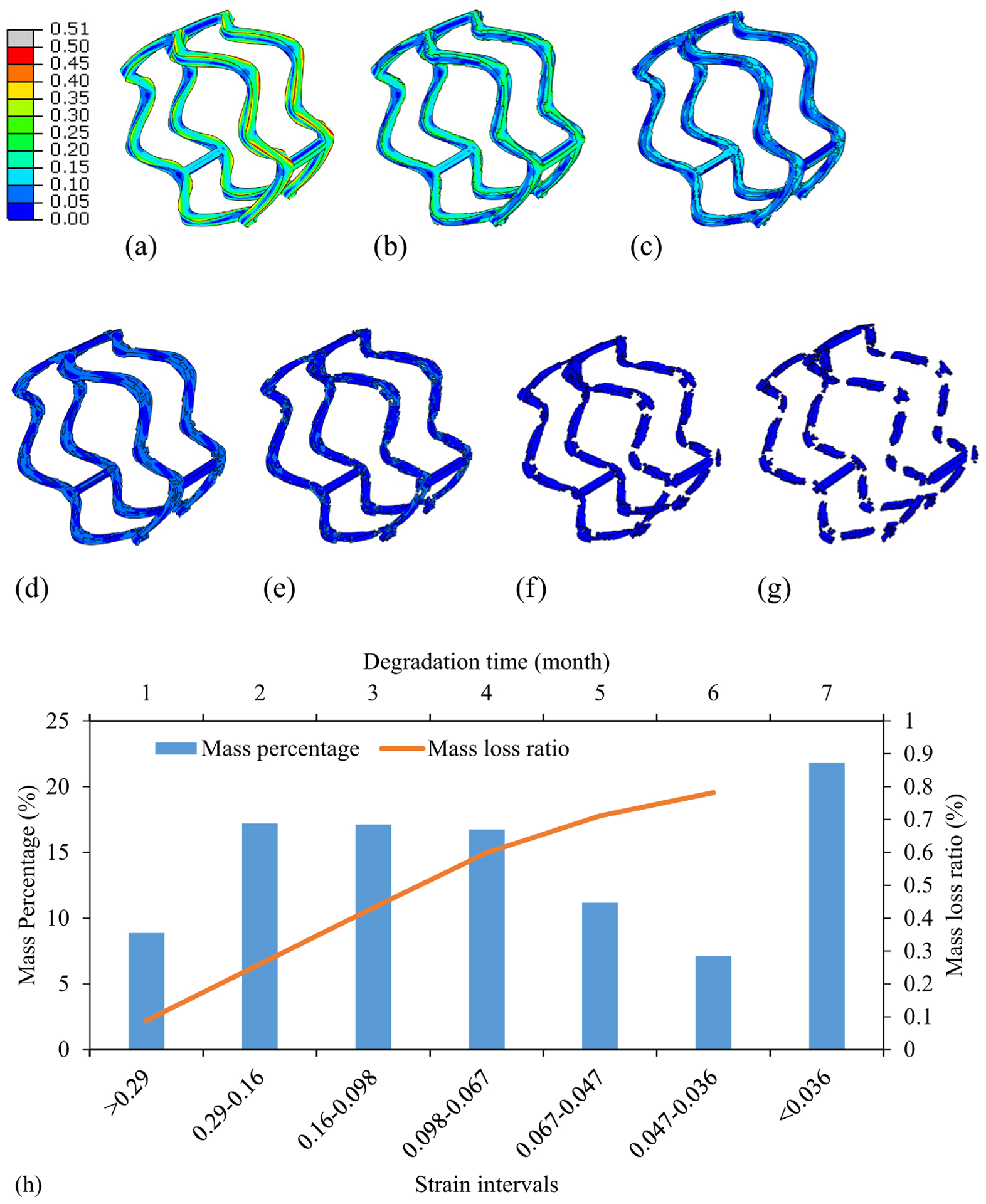
Contour of maximum principal strain at (a) 0 month, (b) first month, (c) second month, (d) third month, (e) fourth month, (f) fifth month, and (g) sixth month. (h) Strain histogram and the mass loss ratio of the stent.

The dynamic degradation behavior of the stent is depicted in Figures 4 and 5. The heterogeneous distribution of the degradation degree (Figure 4a–g) and maximum principal strains (Figure 5a–g) of the PLLA stent was observed throughout the degradation period of 6 months. It is worth noting that the maximum principal strain of the stent remained approximately unchanged during the degradation. During the first 3 months of degradation, the stent kept its mechanical integrity, even the degradation appeared at the outer surface of the stent. As the degradation process evolves, severe degradation took place at the connection struts at the fourth month. The material death technique was adopted to mimic the material absorption, i.e., eliminate the elements that have exceeded the fracture strain. This also resulted in the stent strut thinning. The ring structure of the stent began to break (Figures 4e and 5e) and completely lose its structure integrity at the sixth month (Figures 4f and 5g).

Six representative elements are chosen to better elucidate the degradation process (Figure 4h). It is clear that the maximum principal strains of these representative elements kept approximately at the same magnitude regardless of the degradation. However, the fracture strain decreased nonlinearly down to the value of the maximum principal strain. Specifically, the fracture strain decreased faster in the elements with higher strain values. As a result, the stent region with larger maximum principal strain degraded faster than the one with lower magnitude. In addition, the relationship between the maximum principal strain and the degradation time for initiating the material fracture was highlighted as the dashed line. This further supported that the dynamic degradation behavior of the stent could be predicted based on its strain map.

Both the strain histogram and the mass loss ratio of the stent during degradation process are depicted in Figure 5h. The strain intervals chosen correspond to the strain magnitude at six representative points in Figure 4h. The mass percentage of the elements at different strain intervals contributed to the mass loss at each month. The mass loss ratio is calculated as the mass of absorbed materials over the total mass of the stent. It was observed that the mass loss ratio initiated at the fifteenth day and reached to 78.2% at the sixth month. The mass loss ratio increased linearly with the degradation time during first 4 months. This linear relationship was attributed to approximately equal mass percentage for strain intervals of 0.29–0.16, 0.16–0.098, and 0.098–0.067. The lower mass percentage for strain interval of 0.067–0.047 and 0.047–0.036 contributes to a slightly reduced slope of the mass loss ratio.

**Figure 6:**
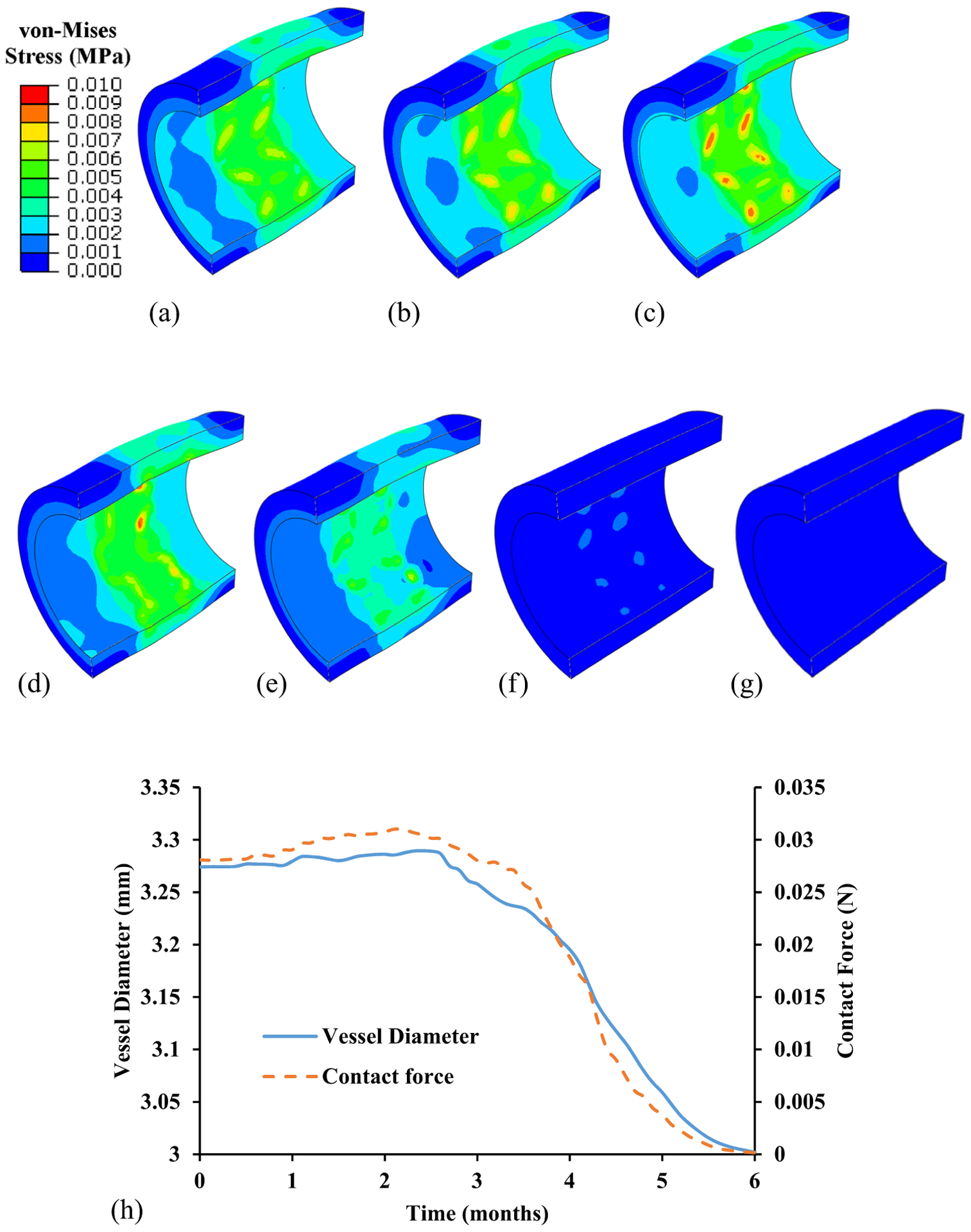
Dynamic stent–artery interaction during degradation. (a–g) Contour of von-Mises stress of artery at (a) 0 month, (b) first month, (c) second month, (d) third month, (e) fourth month, (f) fifth month, and (g) sixth month. (h) Change in vessel diameter and contact force.

The dynamic stent–artery interaction, in terms of the von-Mises stress distribution in the artery, the artery diameter, and the contact force during the degradation process, is depicted in Figure 6. It is clear that higher stresses were observed at the inner surface of the artery and the contact region with stent struts. The peak von-Mises stress increased slightly from 7.8 kPa at 0 month to 11 kPa at the third month. However, the stress pattern remains unchanged until the fourth month. Then the stress pattern blurred and the stent began to lose contact with the artery. At the same time, the artery diameter decreases from 3.28 mm at 0 month to 3.26 mm at the third month, and then to 3mm at the sixth month. This reduction in diameter is associated with the reduced contact force between the stent and the artery (Figure 6h). The reduced contact force/artery diameter indicated the loss of its mechanical integrity as well as its scaffold capacity.

## 4 Discussion

The PLLA BVS is gaining more attention because of its scaffolding capacity and appropriate degradation properties [5,7]. Its degradation progress has been associated with the time and mechanical strains [14,15,24]. In this work, we have developed a strain-based degradation framework with focus on the dynamic interactions between the BVS and the artery during the degradation process. The degradation model was adopted from the literature [15] and implemented into the commercial finite element codes using a user subroutine VUSDFLD and element death technique. The fracture strain of the PLLA material was modulated by the degradation degree, which is a function of the time and local maximal principal strain. The published experimental data [15] were used to estimate the coefficients in the degradation equation and to validate the simulated degradation process.

Before degradation, the detailed implantation procedure of the Absorb GT1 Bioresorbable PLLA stent, i.e., crimping, elastic relaxation, expansion within the artery, and elastic recoil, was simulated. The elastic recoil rate was estimated as 6.3%, which is within the range of expected rate [25]. Stent crimping induced the residual strain in the elastoplastic stent, which affected its strain distribution following expansion [26] and degradation process [27]. In addition, nonuniform strain distribution of the stent was observed. Higher strains occurred on the crowns of the ring strut, which is consistent with the observations from literature [25,28–31].

The heterogeneous degradation in the stent was observed, corresponding to its maximum principal strain distribution. The PLLA material degraded faster at the locations with higher strains, i.e., crowns of the ring strut as well as its connection with the link struts, leading to strut thinning first and then the fracture. The fracture locations match with the reported experiential observations [15]. We have also observed a nonlinear relationship between the maximum principal strain of the stent and the degradation time at fracture (Figure 4g). Specifically, it took less than 1 month for the local fracture at locations with strains larger than 0.3, whereas longer than 7 months at locations with the strain magnitude of 0.02 (equations (2) and (3)). It is worth noting that the strains of the stent exhibited minimal alternations during the degradation process. This implied that the PLLA stent degradation could be predicted based on the strain distribution after its acute deployment. In addition, we can use the stent-induced strain histogram to predict or regulate the mass loss process of the stent (Figure 5h). The mass loss ratio of the stent was attributed to the mass percentage of the stent at various strain intervals. Our stent degradation model results have exhibited a mass loss ratio of 43.2% at the third month and 78.2% at the sixth month. In the corresponding experiments [15], the decrease in number average molecular weight of the stent was reported as 42.3% at the third month and 58.8% at the sixth month.

The contact force between the stent and the artery, i.e., the scaffold capacity of the stent, was reduced during the degradation. Its reduction indicated the loss of structural integrity of the stent. During the first 3 months, strut thinning was observed in the stent with a mass loss ratio of 43.2%, but its structural integrity remained unchanged, which enabled sufficient support for the arterial wall. Following this, severe degradation at the crowns of the ring struts resulted in the noticeable loss of the mechanical integrity and the reduced lumen diameter. The lumen diameter of artery is consistent with the contact force of the stent, as expected. The loss of structural integrity, i.e., strut discontinuity was speculated to be associated with the scaffold collapse and subsequently increased thrombosis rate [32], which needs to be further evaluated.

In the clinical settings, the deployment of the BVS was performed using sequential expansions, recommended by the manufacturer. Instead of the continuous expansion of the traditional stents (a steady pressure over 20–30 s), the deployment of the BVS is performed using sequential expansions with the rule of thumb for increasing in 2 atm after every expansion until the nominal diameter is achieved. It has been shown that the stepwise expansion of stents could achieve a larger lumen than the continuous expansion [33]. These stent expansion patterns could be addressed in our future studies by considering the viscoelastic behavior of the artery and BVS [34].

The hyperelastic PLLA material softening was speculated as one attribute of the PLLA stent degradation [14,35]. It was not explicitly included in this work because the adopted experiential data do not support this observation [15]. The arterial remodeling was also speculated to affect the degradation process. Its role on the stent degradation could be included in our future work if quantitative experimental data are available. The mechanical properties and degradation behavior of the PLLA material could be influenced by its crystallinity degree, which was associated with the fabrication techniques [5,36,37]. This could be potentially used to regulate its degradation time. The discrete experimental data could be enhanced with more time points as well as improved benchtop measurements in the artery, such as the lumen area and arterial strain pattern during the degradation process. The new experimental dataset could reinforce our current computational framework on mimicking the PLLA stent degradation process for better inspecting the underlying mechanobiological processes and enhancing the clinical management of vascular diseases.

## 5 Conclusion

In this work, the strain-based degradation framework was developed to study the dynamic interaction between the BVS and coronary artery throughout the degradation process. The heterogeneous degradation of the stent was attributed to its strain distribution. Degradation-induced strut thinning and discontinuity contributed to the mass loss of the stent, and the reduced contact force between the stent and artery. The nonlinear relationship between the maximum principal strain of the stent and its fracture time was obtained, which could be used to predict the degradation process of the PLLA stent in different mechanical environments. The developed computational model provided more insights into the degradation process, which could complement the discrete experimental data for improving the design and clinical management of biodegradable stents.

## Figures and Tables

**Table 1:** Coefficients of the degradation constitutive model

Coefficients	*a*	*b*	*c*	*m*	*n*
Value	0.385	0.152	0.616	0.342	0.236

**Table 2:** Hyperelastic coefficients of the artery model

Coefficients	*C*_10_	*C*_20_	*C*_30_	*C*_40_	*C*_50_	*C*_60_
Value	6.52 × 10^−3^	4.89 × 10^−2^	9.26 × 10^−3^	0.76	−0.43	8.69 × 10^−2^
